# Evaluation of Immune Responses Induced by Simultaneous Inoculations of Soybean (*Glycine max* [L.] Merr.) with Soil Bacteria and Rhizobia

**DOI:** 10.1264/jsme2.ME18110

**Published:** 2019-02-05

**Authors:** Sayed Ziauddin Hashami, Hiroyuki Nakamura, Naoko Ohkama-Ohtsu, Katsuhiro Kojima, Salem Djedidi, Izumi Fukuhara, Mohammad Daud Haidari, Hitoshi Sekimoto, Tadashi Yokoyama

**Affiliations:** 1 The United Graduate School of Agriculture, Tokyo University of Agriculture and Technology (TUAT) 3–5–8 Saiwai-cho, Fuchu, Tokyo 183–8509 Japan; 2 Faculty of Agriculture, TUAT 3–5–8 Saiwai-cho, Fuchu, Tokyo 183–8509 Japan; 3 Institute of Agriculture, TUAT 3–5–8 Saiwai-cho, Fuchu, Tokyo 183–8509 Japan; 4 Faculty of Agriculture, Utsunomiya University 7–1–2 Yoto, Utsunomiya 321–8585 Japan; 5 Faculty of Agriculture, Kabul University Kabul Afghanistan

**Keywords:** pathogenesis-related proteins, super-nodulation soybean, *Bradyrhizobium diazoefficiens* USDA 110^T^, soil bacteria, plant immune response

## Abstract

Legumes form root nodules and fix atmospheric nitrogen by establishing symbiosis with rhizobia. However, excessive root nodules are harmful to plants because of the resulting overconsumption of energy from photosynthates. The delay of an inoculation of the soybean super-nodulation mutant NOD1–3 with *Bradyrhizobium diazoefficiens* USDA110^T^ by 5 d after an inoculation with several soil bacteria confirmed that one bacterial group significantly decreased root nodules throughout the study period. Moreover, no significant changes were observed in nitrogen fixation by root nodules between an inoculation with USDA 110^T^ only and co-inoculation treatments. To clarify the potential involvement of PR proteins in the restriction of nodule formation in the plants tested, the relative expression levels of *PR-1*, *PR-2*, *PR-5*, and *PDF1.2* in NOD1–3 roots were measured using real-time PCR. One group of soil bacteria (Gr.3), which markedly reduced nodule numbers, significantly induced the expression of *PR-1*, *PR-5* and *PDF1.2* genes by day 5 after the inoculation. By days 7, 10, and 20 after the inoculation, the expression levels of *PR-2* and *PR-5* were lower than those with the uninoculated treatment. Inoculations with this group of soil bacteria resulted in lower root nodule numbers than with other tested soil bacteria exerting weak inhibitory effects on nodulation, and were accompanied by the induction of plant defense-related genes. Thus, *PR* genes appear to play important roles in the mechanisms that suppresses nodule formation on soybean roots.

Legumes form root nodules and fix atmospheric nitrogen (N) by establishing symbiosis with soil bacteria, referred to as rhizobia (29, 30, 35). However, excessive root nodules are harmful to plants because they result in the overconsumption of energy from photosynthates (12, 20, 29). Long-distance signaling (the autoregulation of nodulation) may result in nodule formation on an infected root systemically being suppressed by a subsequently infected root (4, 6, 13).

The production of pathogenesis-related (PR) proteins in plants is very important because they increase whole-plant resistance against a pathogenic attack (24). Several functions and properties of PR proteins were discovered by Van Loon and Van Strien (32). Chitinases and β-1,3-glucanases may be the most important proteins that are abundant in various plant species after a pathogenic attack (10). PR-1 is a dominant group of PR proteins induced by pathogens or salicylic acid (SA) and, since their discovery in 1970, a number of PR-1 proteins have been identified in plants (15). These PR-1 proteins, with molecular weights of between 14 and 17 kDa, are regarded as typical plant systemic acquired resistance markers (1, 23). The suppressive effects of SA on root nodulation were previously reported by Stacey *et al*. (28). Niderman *et al*. (18) demonstrated the antifungal activity of the PR-1 protein at the micromolar level against a number of plant pathogenic fungi. The application of *Bacillus cereus* AR156 significantly reduced the incidence of plant disease by activating induced systemic resistance (ISR) (19). ISR was also activated in a timely manner by the enhanced expression of *PR-1* (19). The PR-2 protein group, which use similar molecular mechanisms to those of β-1,3-glucanases (β-1,3-Gs), includes large and complex gene families that are involved in the plant pathogen defense system as well as other normal developmental processes (1). These proteins have molecular masses of between 33 and 44 kDa (10, 11). The resistivity of β-1,3-glucanase enzymes against various fungi has been reported in many different plant varieties (22). PR-5 represents another type of PR protein that exhibits high antifungal activity levels. They are thaumatin-like proteins that are typically absent in healthy plants, but are expressed exclusively in response to pathogen attacks (17). However, the exact modes of action of these proteins in plants remain unknown. A study by Laurence *et al*. (14) confirmed the antifungal activities of thaumatin-like proteins. The *PDF1.2* gene encodes a member of a group of plant defensins exhibiting antimicrobial activities that are present in all plant species (2). The expression of *PDF1.2* may be induced locally by a pathogen challenge and systematically in inoculated and non-inoculated regions of a plant (21). This activation has been shown to occur through the jasmonate/ethylene-mediated signaling pathway, rather than the SA-dependent pathway (21).

In our laboratory, we have been conducting research to clarify the ecological factors influencing soybean root nodule numbers, with a focus on the potential effects of rhizosphere bacteria on this nodulation process. To exclude the autoregulation mechanism, which is a legume-derived root nodule regulation system, and only examine the influence of rhizosphere bacteria, we conducted the present study using the soybean super-nodulation mutant NOD 1–3 lacking the autoregulation mechanism. Prior to an inoculation with *Bradyrhizobium diazoefficiens* USDA 110^T^, the soybean super-nodulation mutant NOD 1–3 was inoculated independently with four rhizosphere bacteria: *Pseudomonas fluorescens* LRB3W1, isolated from a lettuce rhizosphere (27), *Paenibacillus polymyxa*, isolated from the field at Tokyo University of Agriculture and Technology, *Sinorhizobium meliloti* 1021, isolated from the root nodules of alfalfa, and *Azospirillum* sp. A 205, obtained from a rice rhizosphere paddy field in Thailand.

The numbers of root nodules decreased after the inoculations with these soil bacteria. Furthermore, depending on the microbial species, the extent of the suppression of root nodule numbers differed, and the amounts of methyl jasmonic acid in soybean induced by the inoculated bacteria also varied (unpublished data). Based on an experiment using a split-root system, the pre-inoculation of the soybean super-nodulation mutant NOD1–3 with *P. fluorescens* LRB3W1 resulted in an increase in methyl jasmonic acid concentrations, and, at the same time, the root nodule numbers of the tested plants decreased (unpublished data). Thus, the inoculation of NOD1–3 with various species of soil bacteria resulted in different root nodule numbers. Furthermore, in the split-root system experiment, root nodule numbers may have been influenced by a systemic response induced by the microbial inoculation. This result suggests that plant defense responses, such as systemic acquired resistance and ISR, influence root nodule numbers. The resistance roles of SA and ISR against microbial infections have been previously reported (16, 31).

However, the relationship between soil bacterial species and root nodule numbers in the soybean rhizosphere and the mechanisms by which soil bacteria suppress root nodule numbers in NOD 1–3 plants currently remain unclear. Furthermore, there is limited evidence for a relationship between the suppression of root nodule numbers in NOD1–3 plants and plant defense responses.

In the present study, we co-inoculated the soybean super-nodulation mutant NOD1–3 with individually selected bacterial isolates that exert different effects (non-reducing and reducing) on the root nodule numbers of NOD1–3 and *B. diazoefficiens* USDA 110^T^.

The aims of the present study were: (1) to confirm the effects of co-inoculations with these bacteria together with *B. diazoefficiens* USDA 110^T^ on reductions in nodule numbers in the super-nodulation soybean NOD1–3, and (2) to investigate the effects of single and co-inoculations on the expression levels of the plant defense genes *PR-1*, *PR-2*, *PR-5*, and *PDF1.2*. Our results provide fundamental insights into the host legume’s control of nodulation.

## Materials and Methods

### Bacterial materials

#### Isolation of soil bacteria from soybean roots and rhizospheres from different soils

Eleven soybean plants with their roots and surrounding soil were collected from five different soybean fields, located in Obihiro City on Hokkaido Island, Akita City in Akita Prefecture, Fuchu City in metropolitan Tokyo, Kameoka City in Kyoto Prefecture on Honshu Island, and Saga City in Saga Prefecture on Kyushu Island, Japan. Table 1 shows the sampling locations and soybean cultivars collected. Extra soil was removed from soybean roots by shaking, and roots were placed in medium bottles containing 300 mL of sterilized water for 20 min. The roots were cut into moderately sized portions with scissors, placed in new medium bottles containing 300 mL of sterilized water, and shaken at 100 rpm for 10 min. Shaken solutions were used as soybean rhizosphere soil. These solutions, containing a final concentration of 15% glycerin, were kept at −80°C until used. The remaining roots were placed in 50-mL Falcon tubes, and the root surface of each sample was sterilized with 5% hypochlorous acid for 5 min. Thereafter, to remove hypochlorous acid, surface-sterilized roots were washed with sterile water five times. The roots were cut with scissors and then ground in 20 mL of a 15%-glycerin solution with quartz sand in a mortar. The ground materials were stored as root microbial samples at −80°C. Each soil bacteria sample was diluted with sterilized water to concentrations of 10^−3^ to 10^−6^ and added to nutrient medium plates containing different supplements; King’s A and B (Eiken Chemical, Tochigi, Japan), trypticase soy (Becton Dickinson, Sparks, France), and yeast mannitol; K_2_ HPO_4_ 0.5 g L^−1^, Mg SO_4_·7H_2_O 0.2 g L^−1^, NaCl 0.1 g L^−1^, mannitol 5.0 g L^−1^, sodium gluconate 5.0 g L^−1^, and yeast extract 0.2 g L^−1^ with 1.8% agar (33). Fifty microliters of each sample was transferred to different plates and cultured for 2 d. Colonies of different colors and shapes were selected, transferred to slant media, and then cultured again for 2 d. They were then stored at 4°C until used. A total of 350 isolates were obtained, 92 of which were randomly selected and used in the subsequent experiment shown in Table 2.

### First screening of effects of soil bacteria on root nodulation

In this experiment, 92 isolates and *B. diazoefficiens* USDA 110^T^ were used. One loopful of bacterial cells from each isolate was taken from its slant, and cells were spread on the whole surface of nutrient agar plates and cultured at 28°C for 2 d. Five milliliters of N-free solution containing: CaCl_2_·2H_2_O 294.1 g L^−1^, KH_2_PO_4_ 136.1 g L^−1^, Fe-EDTA 8.4 g L^−1^, MgSO_4_·7H_2_O 123.3 g L^−1^, K_2_SO_4_ 87.0 g L^−1^, MnSO_4_·5H_2_O 0.481 g L^−1^, H_3_BO_3_ 0.247 g L^−1^, ZnSO_4_·7H_2_O 0.288 g L^−1^, CuSO_4_·5H_2_O 0.100 g L^−1^, CoSO_4_·7H_2_O 0.056 g L^−1^, and Na_2_. MoO_4_·2H_2_O 0.056 g L^−1^, pH 6.8 as described by Broughton and Dilworth (3), was added to each plate, the colonies were sufficiently suspended using a platinum loop, and whole suspensions were then mixed with 35 mL of N-free culture solution as inoculum sources. The source of the inoculum from each isolate had approximately 10^4^–10^7^ colony-forming units (CFU) mL^−1^. *B. diazoefficiens* USDA 110^T^ was cultured in yeast-mannitol broth (26) with gentle shaking (80 rpm) at 26°C for 5 d. The culture was centrifuged at 10,000×*g* at 4°C for 5 min. After removing the supernatant, the precipitate was re-suspended in saline and centrifuged again under the same conditions. After centrifugation, the precipitate was re-suspended in an N-free plant culture (3), and a 10^7^ cells mL^−1^ suspension in N-free solution was prepared as an inoculant solution of USDA 110^T^.

In the plant culture, 150 g of sterilized vermiculite (121°C, 0.2 MPa for 20 min) was mixed with 90 mL of N-free solution (3, 26), corresponding to a moisture level of approximately 60%, and loaded into 300-mL autoclaved plant boxes. Seeds of the super-nodulation mutant NOD1–3, a mutant of *Glycine max* (L.) Merr. cv. Williams, were surface sterilized with 3% sodium hypochlorite and germinated for 3 d using sterile Petri plates and sterilized paper towels under dark conditions in a 25°C incubator. Two surface-sterilized NOD 1–3 seeds were then placed in each plant box, and the appropriate amount of N-free solution was added to maintain a moisture level of 60%. The inoculation of the seeds was performed as follows: 3 d after sowing, 92 soil bacterial inocula were applied, and 5 d after the first inoculation, an additional inoculation with the USDA 110^T^ strain was conducted. Two weeks after the USDA 110^T^ inoculation, NOD1–3 roots were carefully collected. The experiment was performed in duplicate in a growth chamber under the following conditions; a 16-h light/8-h dark photoperiod at 25°C/18°C day/night.

In accordance with Yamaya and Arima (34), the root nodule developmental stages were classified as follows: Stage 1 (St1), the meristem had formed, but no cortical swelling was observed; Stage 2 (St2), the meristem showed root cortical swelling, but there was no observable constriction; and Stage 3 (St3) or mature nodules, which clearly showed stricture at the root nodule connection. After the detachment and counting of St3 root nodules, roots were fixed in formaldehyde: acetic acid: 70% (v/v) ethyl alcohol (5:5:90 [v/v/v]) and stained with 0.03% (w/v) toluidine blue solution. Thereafter, root nodule primordial St1 and St2 were observed under an optic microscope.

### Reconfirmation of effects of soil bacteria on root nodule formation

The 92 soil bacteria tested in the first experiment were classified into three groups: Gr.1, Gr.2, and Gr.3, based on their non-reducing and reducing effects on nodulation. Among Gr.1, isolate No. 40, 23, 18, 30, and 39 were selected and found to exert negligible effects on root nodulation. Among Gr.2, the isolates selected were No. 22, 44, 3, 52, and 51, which exerted moderate effects on nodulation. Among Gr.3, which included isolates with strong inhibitory effects on nodulation, isolate No. 71, 14, 80, 57, and 5 were selected. To confirm the effects of the selected isolates on root nodule numbers, the same experiment as that described in section 2.1.2 was performed in quadruplicate.

### Characterization of Gr.1 and Gr.3 isolates to clarify their inoculation effects on root nodule numbers

The root nodule bacterium *B. diazoefficiens* USDA 110^T^ and selected isolates from Gr.1 and Gr.3 were used in this experiment. As shown in Table 2, isolate No. 23, 30, 57, and 71 were obtained from soybean rhizospheric soil and isolate No. 40 and 80 from soybean roots. USDA 110^T^ was cultured in yeast–mannitol broth and shaken in an incubator under dark conditions at 118 rpm at 25°C for 5 d. Gr.1 and Gr.3 soil bacteria were cultured either in King’s media (King A for isolate No. 40 and 80 and King B for isolate No. 23 and 30) or trypticase soy broth for isolate No. 57 and 71 with shaking for more than 2 d. Bacterial cells were collected by centrifugation at 10,000×*g* at 4°C for 10 min twice and then washed twice with 1× TNE buffer; 10 mM Tris, 0.1 M NaCl, and 1 mM EDTA, pH 8 (7). CFUs were adjusted to 10^7^ CFU mL^−1^ using a Coulter Machine (Z1 Coulter Particle Counter; Beckman Coulter, Tokyo, Japan) prior to the inoculation of plants.

### Plant materials

#### Physiological analyses

**Fig. 1:**
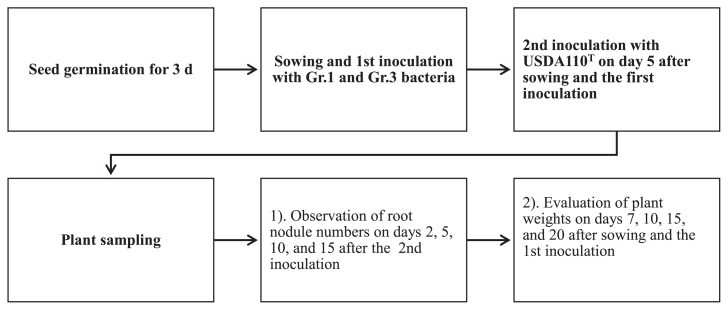
Soybean mutant NOD1–3 inoculation method and sampling for physiological analyses.

Seeds of the super-nodulation mutant NOD1–3, a mutant of *Glycine max* (L.) Merr. cv. Williams, were surface sterilized with 3% sodium hypochlorite and germinated for 3 d using sterile Petri plates and sterilized paper towels under dark conditions in a 25°C incubator. The 300-mL glass jars containing sterilized vermiculite were supplied with a 60% moisture level of N-free nutrient solution (3, 26). After planting seeds (two seeds per jar), Gr.1 and Gr.3 soil bacteria inoculant cells (20 mL) at a density of 10^7^ CFU mL^−1^ were independently applied to the seeds in the jars. The jars were then transferred to a growth chamber and kept under controlled conditions (a 16-h light/8-h dark photoperiod at 25°C/18°C day/night). As shown in Fig. 1, 5 d after planting and the inoculation with Gr.1 or Gr.3 soil bacteria, USDA 110^T^ was either delay-inoculated with soil bacteria to develop root nodules or by itself as a control treatment. This experiment consisted of a completely randomized design with three blocked replicates. Plants were cultured for 20 d, and several measurements were taken at the designated sampling time points (Fig. 1). Root nodule numbers were evaluated 2, 5, 10, and 15 d after the rhizobium (USDA 110^T^) inoculation (DAI), and acetylene reduction assays of these root nodules were assessed on 5, 10, and 15 DAI with rhizobia. We also measured plant weights (fresh and dry) 7, 10, 15, and 20 d after sowing and the inoculation (DAS). In acetylene reduction assays, fresh roots that contained root nodules were placed in 300-mL glass jars, the air in the jar was supplemented with 10% acetylene (v/v) for each treatment, and the jars were incubated in an incubator (25°C) for 1 h. The ethylene concentration in each jar was measured using a gas chromatograph (Shimadzu 2014 AF, Kyoto, Japan).

### Evaluation of root nodule primordia

Root primordia and root nodules were counted using the method described by Yamaya and Arima (34). The discrimination among root nodule developmental stages was described in section 2.1.2.

### RNA extraction from NOD1–3 soybean roots

A second experiment was conducted concurrently under similar conditions, as described in section 2.2.1, using the super-nodulation soybean NOD1–3. However, in this experiment, we used 100-mL capacity cell trays instead of 300-mL glass jars, and USDA 110^T^ was either inoculated alone at planting or delay-inoculated on day 5 after the inoculation with Gr.1 and Gr.3 soil bacteria. In this experiment, there were four sets of treatments: 1) a treatment without the bacterial inoculation, control 1 (Ctr1); 2) USDA 110^T^ only, control 2 (Ctr2); 3) Gr.1 and Gr.3 soil bacteria only; and 4) a co-inoculation of USDA 110^T^ with Gr.1 and Gr.3 soil bacteria (Gr.1/Gr.3+USDA 110^T^). Portions of the main roots, including the lateral roots, were collected for RNA isolation at the designated time points of 2, 5, 7, 10, 15, and 20 DAS. At each sampling time point, the sampled roots were immediately placed in liquid N and then stored at −80°C until total RNA was extracted. Total RNA was isolated using RNAiso Plus reagent (Takara Bio, Kusatsu, Japan) according to the manufacturer’s instructions. Qualitative and quantitative characterizations of RNA samples were conducted using a NanoDrop 1000 Spectrophotometer (Thermo Fisher Scientific, Wilmington, DE, USA), and samples were then stored at −80°C for gene expression analyses.

### Synthesis of cDNA for real-time PCR analyses

Samples corresponding to the 2-, 5-, 7-, 10-, and 20-d time points were selected for cDNA synthesis and real-time PCR. In brief, RNA samples were treated with DNaseI (Takara Bio) and reverse-transcribed using the PrimeScript^TM^ RT reagent Kit (Perfect Real Time) with gDNA Eraser (Takara Bio) and oligo (dT)_20_ according to the manufacturer’s instructions. Reverse-transcribed cDNA from 1 μg of RNA was used as the template for real-time PCR. The real-time PCR analysis was conducted using a LightCycler^®^ Nano System (Roche Diagnostics, Basel, Switzerland; https://lifescience.roche.com/shop/home) and LightCycler^®^ FastStart Essential DNA Green Master (Roche Diagnostics) as recommended by the manufacturer. The primer sets used in real-time PCR are listed in Table S1. *SUBI-2* (ubiquitin) was used as an internal control gene, and sample cycle threshold (CT) values were normalized for each template using the reference gene as the control. The 2^−ΔΔCT^ method was then performed to analyze relative changes in gene expression. Three independent biological replicates of each treatment were used in a single quantitative real-time-PCR reaction for statistical analyses.

## Results

### Evaluation of effects of soil bacteria on root nodulation in terms of root nodule numbers

**Fig. 2:**
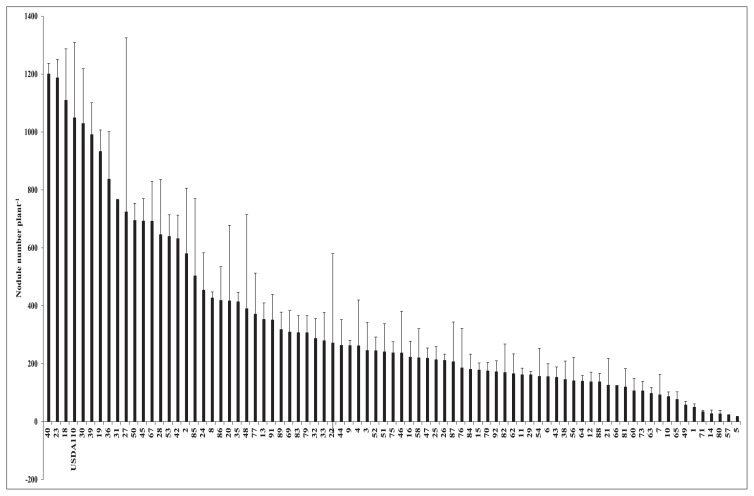
Soybean mutant NOD1–3 total root nodule number (stage 1+stage 2+stage 3) under a single/co-inoculation with *Bradyrhizobium diazoefficiens* USDA 110^T^ and different soil bacteria.

Fig. 2 shows the influence of pre-inoculations with soil bacteria on root nodulation in the soybean line NOD 1–3. Among the 92 strains used for the first inoculation, prior to the inoculation with *B. diazoefficiens* USDA 110^T^, 12 isolates markedly inhibited the growth of the plant, and, thus, were eliminated from further examinations. Among the remaining 80 strains, some isolates, such as No. 40, 23, and 18, did not change root nodule numbers from those with the control treatment in which there was no pre-inoculation with soil bacteria. However, other isolates, including No. 80, 57, and 5, induced decreases in root nodule numbers that corresponded to 97.4, 97.8, and 98.4%, respectively, those in control plants.

**Fig. 3:**
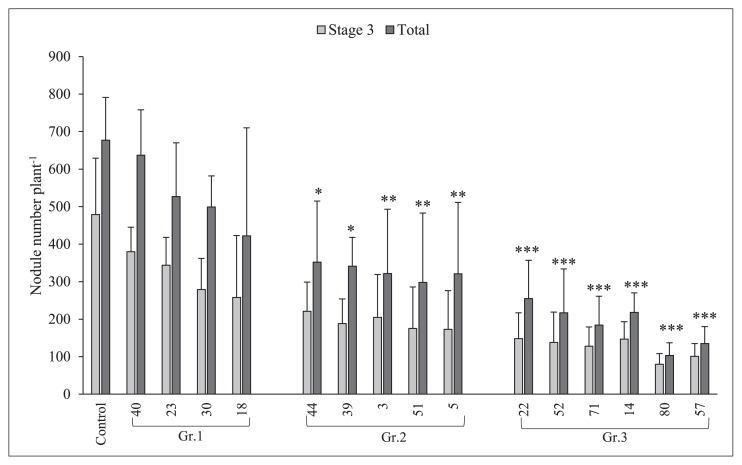
Soybean mutant NOD1–3 stage 3 and total root nodule numbers under a single/co-inoculation with *Bradyrhizobium diazoefficiens* USDA 110^T^ (control) and different soil bacteria. The statistical analysis of data was performed in consideration of the total nodule number using Dunnett’s test comparisons with the control (*n*=4, *P<0.05, **P<0.01, ***P<0.001). Error bars indicate the standard deviations of four replicates.

These results were obtained as the mean and error of two plant samples, and differences were noted when error bars did not overlap with the control. Isolate No. 40, 23, and 18 clearly overlapped with the control, and, thus, were not different. The results of the confirmation test of the effects of the selected soil bacteria on root nodule numbers are shown in Fig. 3. Based on their effects on root nodule numbers, namely, slightly, moderately, and markedly lower nodule numbers than those of the control, these bacteria were placed into three groups: Gr.1, Gr.2, and Gr.3, respectively. The mean reductions induced in root nodule numbers by the isolates of Gr.1, Gr.2, and Gr.3 were 23, 52, and 74%, respectively. In comparison with the results shown in Fig. 2, isolate No. 40, 23, and 18, which resulted in higher root nodule numbers than those of the control, did not increase this parameter in the confirmation test. Additionally, isolate No. 30 reduced root nodule numbers by 27% from that of the control. However, these results were not significantly different from those in Fig. 2. Regarding the remaining 11 isolates, root nodule numbers were significantly lower than those of the control.

### Characterization of Gr.1 and Gr.3 isolates to clarify their inoculation effects on root nodule numbers

Based on the above results, we co-inoculated the super-nodulation soybean NOD1–3 with most of the isolates of Gr.1 and selected representative isolates from Gr.3 with *B. diazoefficiens* USDA 110^T^. These species were identified using a sequence analysis of 16S rRNA genes, and the 16S rRNA gene sequences of the selected isolates have been deposited in the DNA Data Bank of Japan. Isolates from Gr.1 were as follows: No. 23, 30, and 40 corresponding to *Pseudomonas* sp. strain JP-O-23, *Chryseobacterium* sp. strain JP-O-30, and *Agrobacterium tumefaciens* strain JP-M-40, respectively. Isolates from Gr.3 were as follows: No. 57, 71, and 80 corresponding to *Bosea* sp. strain JP-Y-57, *Niabella* sp. strain JP-B-71, and *Bosea* sp. JP-O-80, respectively. GenBank accession numbers for isolates JP-O-23, JP-O-30, JP-M-40, JP-Y-57, JP-B-71, and JP-O-80 are LC388678, LC388676, LC388673, LC388674, LC388677, and LC388675, respectively.

**Fig. 4:**
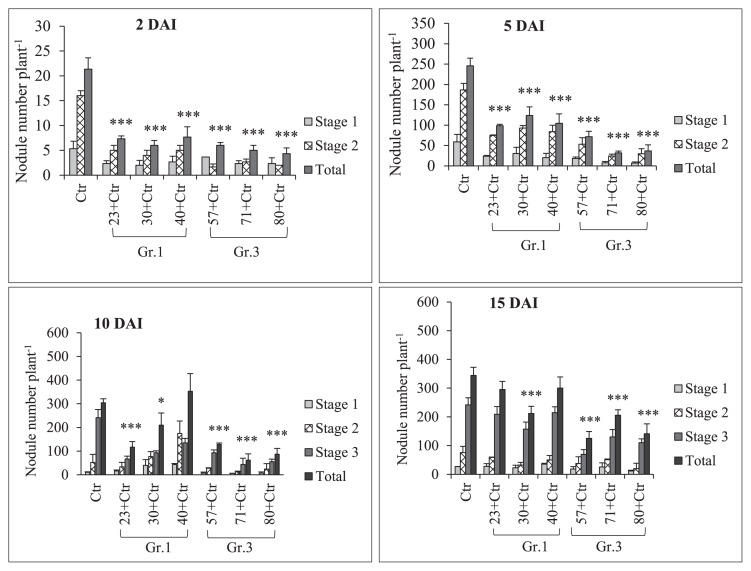
Soybean mutant NOD1–3 root nodule numbers under a single/co-inoculation with *Bradyrhizobium diazoefficiens* USDA 110^T^ (Ctr) and Group (Gr) 1 or 3 soil bacteria. DAI; days after the inoculation with rhizobia. The statistical analysis of data was performed in consideration of the total nodule number using Dunnett’s test comparisons with the control (*n*=3, *P<0.05, ***P<0.001). Error bars indicate the standard deviations of three replicates.

**Fig. 5:**
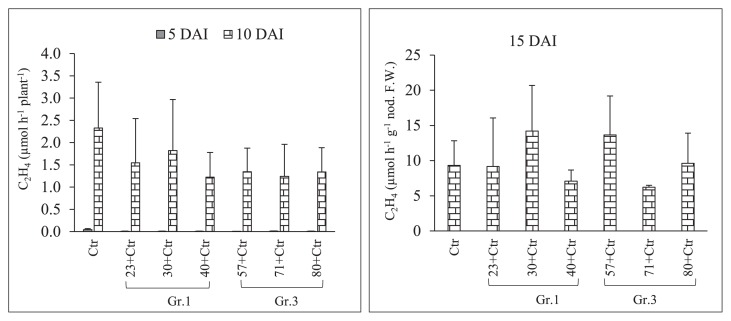
Acetylene reduction activity (ARA) of soybean mutant NOD1–3 root nodules in response to a single/co-inoculation with *Bradyrhizobium diazoefficiens* USDA 110^T^ (Ctr) and Group (Gr) 1 or 3 soil bacteria. DAI; days after the inoculation with rhizobia, and error bars indicate the standard deviations of three replicates.

**Fig. 6:**
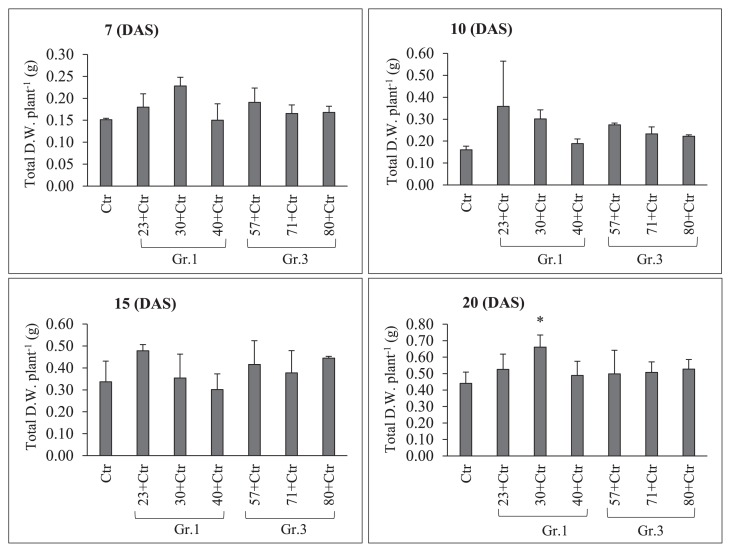
Total plant dry weight (g) as influenced by a single/co-inoculation of *Bradyrhizobium diazoefficiens* USDA 110^T^ and Group (Gr) 1 or 3 soil bacteria. Statistical analyses were performed in comparison with the control. (Dunnett test, *P<0.05, *n*=3). Error bars indicate the standard deviations of three replicates. DAS; days after sowing.

Sampling to observe root nodule numbers was performed on 2, 5, 10, and 15 DAI with *Rhizobium*. The results of pre-inoculations with Gr.1 and Gr.3 soil bacteria on the root nodulation of the soybean line NOD 1–3 are shown in Fig. 4 and Table 3. The pre-inoculation with Gr.1 and Gr.3 isolates inhibited the number of primordial root nodules that formed (St1+St2) significantly more than the control treatment (USDA 110^T^) on 2 and 5 DAI with *Rhizobium*. We did not observe any St3 or mature root nodules on these sampling days. However, a decrease in the number of root nodule primordia was observed on 2 DAI, and the difference in the number of root nodule primordia between Gr.1 and Gr.3 was not significant. Furthermore, significantly higher numbers of root nodule primordia were observed in Gr.1 isolates, except for isolate No. 23, than in Gr.3 on day 5 of sampling. Furthermore, isolate No. 57, 71, and 80, belonging to Gr.3, significantly reduced the root nodule numbers of the tested plants to 51, 80, and 71%, respectively, that of the control on 10 DAI. This level of reduction in root nodulation was not observed with the pre-inoculation with G.1 isolates, except for isolate No. 23, which significantly reduced root nodulation by 62%. However, isolate No. 40 of Gr.1 increased the root nodule number by 16% that of the control on day 10 of sampling. Moreover, on day 15, isolate No. 57, 71, and 80 of Gr.3 significantly decreased the formation of root nodules to 64, 40, and 59%, respectively, that of the control treatment, while root nodulation was not significantly altered from that of the control by the pre-inoculation with Gr.1 isolates at this sampling time point. Thus, root nodule formation in the super-nodulation soybean NOD1–3 by the pre-inoculation with Gr.3 isolates was significantly reduced from that with the control treatment at all sampling time points. However, no significant differences were observed in N-fixation activity levels between the treatments tested (Fig. 5 and Table 3); however, isolate No. 30, 57, and 80 showed greater N-fixation activity levels than that of the control treatment on 15 DAI with *Rhizobium*. Furthermore, total plant dry weights were not significantly affected by the bacterial inoculation on days 7, 10, 15, and 20 of sampling. However, on day 20, the co-inoculation treatment with isolate No. 30 significantly increased total plant dry weights (Fig. 6).

### Expression analyses of plant defense-related genes using real-time PCR

**Fig. 7:**
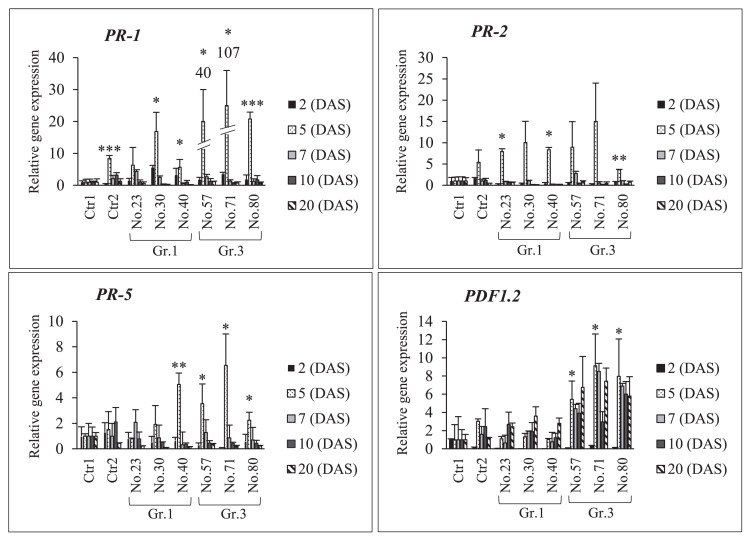
RT-PCR analyses of plant defense-related genes (*PR-1*, *PR-2*, *PR-5*, and *PDF1.2*) in soybean mutant NOD1–3 roots after a single inoculation with *Bradyrhizobium diazoefficiens* USDA 110^T^ and Group (Gr) 1 or 3 soil bacteria. Ctr1, treatment without a bacterial inoculation; and Ctr2, USDA 110^T^ only inoculation. The expression level of each gene was normalized to the *SUBI-2* (ubiquitin) gene. The means±standard deviations of three biological replicates are shown as 1 in the mean of the control (Ctr1) condition. Statistical analyses (Dunnett’s test, *n*=3, *P<0.05, **P<0.01, *** P<0.001) were performed for comparisons with Ctr1.

The results of the relative expression analysis of plant defense genes (*PR-1*, *PR-2*, *PR-5*, and *PDF1.2*) after single inoculations with Gr.1, Gr.3, or USDA 110^T^ in NOD1–3 roots are shown in Fig. 7 and Table S2. Gr.1 isolate No. 30 and 40 increased the expression of the *PR-1* gene by 6- and 3-fold, respectively, that of the control on 2 DAS. Similarly, all of the treatments with Gr.3 isolates induced the expression of *PR-1* at the sampling time points indicated. However, the expression levels of other genes (*PR-2*, *PR-5*, and *PDF1.2*) on 2 DAS were lower than those of Ctr1. Furthermore, all of the Gr.3 isolates increased the expression levels of the *PR-1*, *PR-5*, and *PDF1.2* genes significantly more than Ctr1 on 5 DAS, except for the levels of *PR-2*, after the inoculation with isolate No. 57 and 71. Among the Gr.1 isolates, No. 30 significantly increased the expression level of *PR-1*, while isolate No. 40 increased the expression levels of *PR-1*, *PR-2*, and *PR-5*, but not that of *PDF1.2*, on day 5. On subsequent sampling days, the expression levels of *PR-1*, *PR-2*, and *PR-5* were up- or down-regulated by single inoculations with USDA 110^T^ or Gr.3 soil bacteria. However, the expression level of *PDF1.2* was strongly induced after the inoculation with Gr.3 soil bacteria on subsequent sampling days. For example, isolate No. 57 increased the expression of the *PDF1.2* gene by 4-, 4-, and 7-fold on days 7, 10, and 20 of sampling, respectively. Increases in *PDF 1.2* expression levels on days 7, 10, and 20 of sampling was recorded for isolate No. 71 (by 8-, 3-, and 7-fold, respectively) and No. 80 (by 7-, 6-, and 6-fold, respectively).

**Fig. 8:**
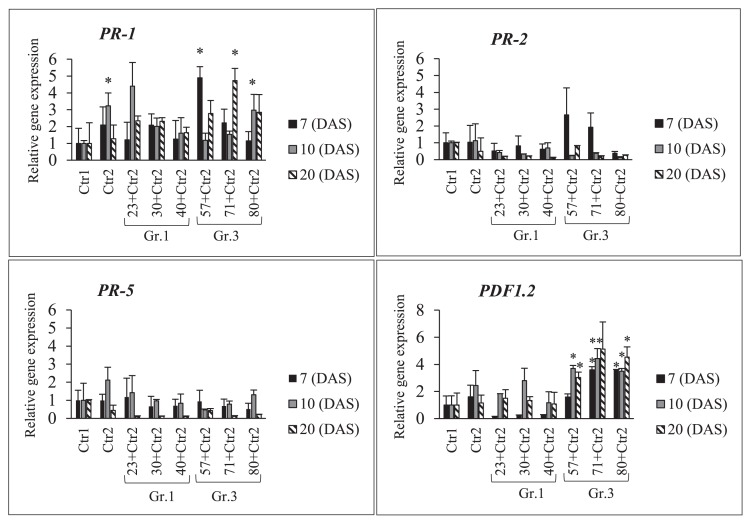
RT-PCR analyses of plant defense-related genes *(PR-1*, *PR-2*, *PR-5*, and *PDF1.2*) in soybean mutant NOD1–3 roots after a co-inoculation with *Bradyrhizobium diazoefficiens* USDA 110^T^ and individual Group (Gr) 1 or 3 soil bacteria. Ctr1, treatment without a bacterial inoculation; and Ctr2, USDA 110^T^ only inoculation. The expression level of each gene was normalized to the *SUBI-2* (ubiquitin) gene. The means±standard deviations of three biological replicates are shown as 1 in the mean of the control (Ctr1) condition. Statistical analyses (Dunnett’s test, *n*=3, *P<0.05, **P<0.01) were performed for comparisons with Ctr1.

The increased expression of the above-described genes in NOD1–3 roots after the co-inoculation with USDA 110^T^ plus individual Gr.1 or Gr.3 isolates is shown in Fig. 8 and Table S3. The co-inoculation treatments with Gr.3 isolates strongly induced the expression of the *PR-1* and *PDF1.2* genes at different sampling time points. For example, isolate No. 57 on day 7, isolate No. 71 on days 10 and 20, and isolate No. 80 on day 20 increased the expression levels of the *PR-1* and *PDF1.2* genes significantly more than the control. Moreover, the expression of the *PR-2* gene was down-regulated by the co-inoculation treatments with Gr.1 and Gr.3 isolates on days 7, 10, and 20, except for isolates No. 57 and 71 of Gr. 3, which induced gene expression on day 7. Similarly, the expression of the *PR-5* gene was down-regulated by co-inoculation treatments with Gr.1 and Gr.3 isolates, except for USDA 110^T^ alone and isolates No. 23 and 80, which more strongly induced gene expression on day 10 than Ctr1. Thus, co-inoculation treatments with Gr.3 isolates more strongly induced the expression of the *PR-1* and *PDF1.2* genes in NOD1–3 roots on days 7, 10, and 20 than the control. However, co-inoculation treatments with Gr.1 isolates did not increase the expression levels of the *PR-2* or *PR-5* gene at any of the sampling time points tested.

## Discussion

### Evaluation of effects of soil bacteria on root nodule numbers

Among the 80 strains evaluated in the present study, 81% exhibited the ability to suppress root nodule numbers by more than 50% (versus the control treatment, Fig. 2) when used as pre-inoculation sources prior to the inoculation of soybean plants with *B. diazoefficiens* USDA 110^T^. No relationship was observed between the effects of these soil bacteria on root nodule numbers and their sampling sites, soybean varieties, or isolate locations (soil or roots). Regarding their effects on nodulation, the inoculation of NOD1–3 with different strains of soil bacteria resulted in differences in root nodule numbers. Depending on the strains used, they exerted three types of effects (slight or no inhibition, moderate inhibition, and marked inhibition) on nodulation versus the control treatment in which these strains were not applied. Furthermore, the results of the confirmation experiment (Fig. 3), in which Gr.1, Gr.2, and Gr.3 soil bacteria were used as the first inoculants, showed that most of the isolates belonging to Gr.2 and all isolates of Gr.3 bacteria reduced the numbers of root nodules significantly more than the control treatment (USDA 110^T^). Thus, pre-inoculations with Gr.2 and Gr.3 induced moderate and marked reductions in root nodule numbers, respectively.

### Characterization of Gr.1 and Gr.3 isolates to clarify their inoculation effects on root nodule numbers

Super-nodulation (or hyper-nodulation) mutant soybean lines form markedly higher numbers of root nodules than their parental lines (5, 25, 29). We observed that the prei-noculation of NOD1–3 roots with Gr.1 and Gr.3 soil bacteria resulted in significantly lower numbers of root nodule primordia than the control treatment (USDA 110^T^) without a soil bacteria pre-inoculation on 2 and 5 DAI with *Rhizobium*. Furthermore, the pre-inoculation with Gr.3 soil bacteria reduced the formation of root nodule primordia and stage 3 (St3) or mature nodules on 10 and 15 DAI with *Rhizobium* significantly more than the control treatment. Pre-inoculations with Gr.1 soil bacteria did not significantly reduce root nodulation on 10 and 15 DAI from that with the control treatment (Fig. 4 and Table 3). These results indicate that depending on the bacterial species, the extent of the suppression of root nodule numbers differed, and further studies are needed to elucidate the effects of these interactions between these soil bacteria, *Rhizobium*, and super-nodulation soybean. At all sampling time points, Gr.3 isolates exerted significantly strong reductive effects on the root nodule numbers of the super-nodulation soybean mutant NOD1–3. These results also confirmed that NOD1–3 root nodule numbers were not significantly reduced at any sampling time points by the pre-inoculation with Gr.1 soil bacteria from that by the control treatment (USDA 110^T^) without a soil bacterial pre-inoculation (Fig. 4 and Table 3). Fewer root nodules formed in NOD1–3 co-inoculated with Gr.1 soil bacteria than with the control. These results suggest that root nodule numbers in the super-nodulation soybean line were suppressed by the delayed inoculation with the root-nodulating bacterium (USDA 110^T^) on 5 DAI. Nodule formation on an infected root systematically suppresses formation on a subsequently infected root (13). Additionally, no significant differences were observed in the N-fixation activity levels of those root nodules among the tested treatments. Moreover, total plant dry weights were significantly higher with the co-inoculation treatment with isolate No. 30 on day 20 than with the control treatment (Fig. 6). A previous study by Dubey (8) showed that the co-inoculation with *B. diazoefficiens* and PGPR microorganisms significantly increased soybean growth and its yield components over that with *B. diazoefficiens* alone. Based on our results and these findings, the combined inoculation with USDA 110^T^ and some soil bacteria resulted in the maximum plant total dry weight, which was significantly higher than that with the USDA 110^T^ inoculation alone.

### Expression analyses of plant defense-related genes using real-time PCR

To clarify the plant immune responses induced in NOD1–3 roots by a single/co-inoculation with different soil bacteria and USDA 110^T^, we evaluated the relative expression levels of the plant defense genes *PR-1*, *PR-2*, *PR-5*, and *PDF1.2* using real-time PCR. The roles of the encoded proteins in the restriction of soybean root nodulation after an inoculation with the rhizobium (USDA 110^T^) and a combined inoculation with USDA 110^T^ and other soil bacteria have not yet been confirmed. The expression levels of *PR-1*, *PR-5*, and *PDF1.2* were significantly increased by the single inoculation of NOD1–3 roots with Gr.3 soil bacteria on 5 DAI. However, these soil bacteria (Gr.3) significantly suppressed NOD1–3 root nodule formation and development at all sampling time points. These results indicate that the suppressive regulation of root nodule formation and development started on day 5 when NOD1–3 roots were inoculated with *Rhizobium*.

The expression levels of *PR-1* and *PDF1.2* were strongly induced by the co-inoculation with USDA 110^T^ and Gr.3 soil bacteria on subsequent sampling days (Fig. 8 and Table S3). The defensive roles of PR proteins and their direct antimicrobial effects in plants have been reported (9, 24). Furthermore, Nie *et al*. (19) found that enhanced PR-1 protein expression encouraged plants to activate ISR against pathogenic bacteria. The defensive roles of PR-2, PR-5, and PDF1.2 against pathogenic bacteria have been reported previously (2, 14, 22).

Isolates of Gr.1 soil bacteria, which produced a higher number of root nodules in NOD1–3 than Gr.3 isolates, did not strongly induce the expression of the *PR-1*, *PR-2*, *PR-5*, and *PDF1.2* genes. According to our nodulation data, a marked difference in the number of NOD1–3 root nodules was observed on day 15 between the co-inoculation treatments with Gr.3 and Gr.1 soil bacteria. Root nodulation was inhibited significantly more by Gr.3 soil bacteria than by the control treatment, and Gr.3 soil bacteria also strongly induced the expression of *PR-1* and *PDF1.2* (Fig. 4 and 8). Similarly, isolate No. 40 induced the formation of root nodules slightly more than the control treatment on days 10 and 15. However, this isolate (No. 40) reduced the expression levels of *PR-1* and *PDF1.2* on days 10 and 20. Thus, the plant defense-related genes *PR-1*, *PR-2*, *PR-5*, and *PDF1.2* appear to play important mechanistic roles in suppressing root nodulation in super-nodulation soybean roots. These results also suggest that a single and co-inoculation with Gr.3 soil bacteria strongly induced the expression of plant defense genes, particularly *PR-1* and *PDF1.2*, which may have led to lower nodulation levels in super-nodulation soybean roots. Thus, inoculations containing Gr.3 soil bacteria resulted in the inhibition of root nodule formation accompanied by the induction of plant defense-related genes.

In conclusion, the present results suggest that various types of bacteria exist in field soils and exert different effects on the nodule formation-related activities of rhizobia. One group of soil bacteria (Gr.3) that markedly reduced root nodule numbers when co-inoculated with USDA 110^T^ also significantly increased the expression levels of *PR-1*, *PR-5*, and *PDF1.2* on 5 DAI. Moreover, other isolates (Gr.1) exerted weaker inhibitory effects on root nodulation than Gr.3 isolates, and these effects were accompanied by the general down-regulation of plant defense-related genes.

To the best of our knowledge, this is the first study to suggest the involvement of PR proteins in the mechanisms suppressing nodule formation in super-nodulation soybean roots. Further studies are needed to elucidate the effects of these interactions between these soil bacteria, *Rhizobium*, and the super-nodulation soybean on the regulation of root nodule formation in order to balance the nutritional requirements of soybean plants.

## Supplementary Information



## Figures and Tables

**Table 1:** Sampling locations and soybean cultivars collected.

Region	Soybean cultivars	Latitude and longitude
Hokkaido	Yumehomare Oosode	42.92° N, 143.20° E
Akita	TZO 1 Ryuhou Enrei	39.72° N, 140.10° E
Tokyo University of Agriculture and Technology, Tokyo	NOD1-3 Williams 82	35.67° N, 139.48° E
Kyoto	Kurodaizu	35.01° N, 135.57° E
Saga	Fukuyutaka Bunny Murayutaka	33.26° N, 130.30° E

**Table 2:** Soil microorganisms isolated from rhizosphere and roots of different soybean cultivars.

Isolate ID	Soybean cultivars	Isolation part	Culture media	Isolate ID	Soybean cultivars	Isolation part	Culture media
No.1	Oosode	Rhizosphere	Trypticase soy	No.47	Murayutaka	Rhizosphere	King A
No.2	Oosode	Rhizosphere	Trypticase soy	No.48	Fukuyutaka	Root	King A
No.3	Oosode	Rhizosphere	Trypticase soy	No.49	TZO 1	Rhizosphere	King A
No.4	Oosode	Root	Trypticase soy	No.50	TZO 1	Rhizosphere	King A
No.5	Oosode	Root	Trypticase soy	No.51	TZO 1	Rhizosphere	King A
No.6	Oosode	Root	Trypticase soy	No.52	TZO 1	Rhizosphere	YMA
No.7	Oosode	Root	Trypticase soy	No.53	TZO 1	Rhizosphere	YMA
No.8	Ryuhou	Rhizosphere	Trypticase soy	No.54	TZO 1	Rhizosphere	YMA
No.9	Ryuhou	Rhizosphere	Trypticase soy	No.55	Yumehomare	Rhizosphere	Trypticase soy
No.10	Ryuhou	Root	Trypticase soy	No.56	Yumehomare	Rhizosphere	Trypticase soy
No.11	Ryuhou	Root	Trypticase soy	No.57	Yumehomare	Rhizosphere	Trypticase soy
No.12	Yumehomare	Rhizosphere	Trypticase soy	No.58	Murayutaka	Rhizosphere	Trypticase soy
No.13	Yumehomare	Root	Trypticase soy	No.59	Murayutaka	Rhizosphere	Trypticase soy
No.14	Fukuyutaka	Rhizosphere	Trypticase soy	No.60	Yumehomare	Rhizosphere	Trypticase soy
No.15	Fukuyutaka	Rhizosphere	Trypticase soy	No.61	Yumehomare	Rhizosphere	Trypticase soy
No.16	Fukuyutaka	Root	Trypticase soy	No.62	Yumehomare	Rhizosphere	Trypticase soy
No.17	Fukuyutaka	Root	Trypticase soy	No.63	Murayutaka	Root	Trypticase soy
No.18	TZO 1	Rhizosphere	King B	No.64	Murayutaka	Root	Trypticase soy
No.19	TZO 1	Rhizosphere	King B	No.65	Murayutaka	Root	Trypticase soy
No.20	TZO 1	Rhizosphere	King B	No.66	Murayutaka	Root	Trypticase soy
No.21	TZO 1	Rhizosphere	King B	No.67	Fukuyutaka	Rhizosphere	Trypticase soy
No.22	TZO 1	Rhizosphere	King B	No.68	Fukuyutaka	Rhizosphere	Trypticase soy
No.23	Oosode	Rhizosphere	King B	No.69	Fukuyutaka	Rhizosphere	Trypticase soy
No.24	Ryuhou	Rhizosphere	King B	No.70	Bunny	Root	Trypticase soy
No.25	Ryuhou	Rhizosphere	King B	No.71	Bunny	Rhizosphere	Trypticase soy
No.26	Ryuhou	Rhizosphere	King B	No.72	Bunny	Rhizosphere	Trypticase soy
No.27	Ryuhou	Rhizosphere	King B	No.73	Bunny	Rhizosphere	Trypticase soy
No.28	Bunny	Root	King B	No.74	Bunny	Rhizosphere	Trypticase soy
No.29	Bunny	Root	King B	No.75	Oosode	Rhizosphere	King A
No.30	Oosode	Rhizosphere	King B	No.76	Oosode	Rhizosphere	King A
No.31	Fukuyutaka	Rhizosphere	King B	No.77	Oosode	Rhizosphere	King A
No.32	Fukuyutaka	Rhizosphere	King B	No.78	Oosode	Root	King A
No.33	Fukuyutaka	Rhizosphere	King B	No.79	Oosode	Root	King A
No.34	Bunny	Rhizosphere	King A	No.80	Oosode	Root	King A
No.35	Bunny	Root	King A	No.81	Yumehomare	Rhizosphere	King A
No.36	Bunny	Root	King A	No.82	Yumehomare	Rhizosphere	King A
No.37	Bunny	Root	King A	No.83	Yumehomare	Rhizosphere	King A
No.38	Murayutaka	Root	King A	No.84	Murayutaka	Rhizosphere	King B
No.39	Murayutaka	Root	King A	No.85	Yumehomare	Rhizosphere	King B
No.40	Murayutaka	Root	King A	No.86	Yumehomare	Rhizosphere	King B
No.41	Murayutaka	Root	King A	No.87	Yumehomare	Rhizosphere	King B
No.42	Murayutaka	Root	King A	No.88	Yumehomare	Rhizosphere	King B
No.43	Murayutaka	Root	King A	No.89	Oosode	Root	King B
No.44	Murayutaka	Root	King A	No.90	Oosode	Root	King B
No.45	Murayutaka	Rhizosphere	King A	No.91	Oosode	Root	King B
No.46	Murayutaka	Rhizosphere	King A	No.92	Oosode	Root	King B

**Table 3:** Soybean mutant NOD1-3 root nodule numbers and acetylene reduction assay (ARA) results as affected by a single/co-inoculation with *Bradyrhizobium diazoefficiens* USDA 110^T^ (control) with Group (Gr) 1 (No.23, 30, and 40) or 3 (No.57, 71, and 80) soil bacteria.

Sampling (d)		Treatments	Root nodule numbers/plant				C_2_H_4_ (μmol h^−1^ plant^−1^)

			Stage 1	Stage 2	Stage 3	Total	
2 (DAI)		Control	5	16	NO	21±2	ND
		No.23+control	2	5	NO	7±1***	ND
	Gr.1	No.30+control	2	4	NO	6±1***	ND
		No.40+control	3	5	NO	8±2***	ND
		No.57+control	4	2	NO	6±1***	ND
	Gr.3	No.71+control	2	3	NO	5±1***	ND
		No.80+control	2	2	NO	4±1***	ND

5 (DAI)		Control	59	187	NO	246±2	0.045±0.026
		No.23+control	24	75	NO	99±3***	0.009±0.001
	Gr.1	No.30+control	31	93	NO	24±2***	0.008±0.003
		No.40+control	20	84	NO	105±2***	0.009±0.002
		No.57+control	18	53	NO	72±1***	0.008±0.001
	Gr.3	No.71+control	8	24	NO	32±4***	0.012±0.003
		No.80+control	7	30	NO	37±1***	0.009±0.001

10 (DAI)		Control	11	52	242	304±17.0	0.045±0.026
		No.23+control	16	34	67	117±23.6***	0.009±0.001
	Gr.1	No.30+control	40	77	93	210±50.9*	0.008±0.003
		No.40+control	43	175	135	353±75.1	0.009±0.002
		No.57+control	8	27	93	128±5.7***	0.008±0.001
	Gr.3	No.71+control	6	13	44	62±26.1***	0.012±0.003
		No.80+control	8	24	55	87±24.7***	0.009±0.001

15 (DAI)		Control	27	76	242	344±28.4	9.3±3.5
		No.23+control	28	59	209	295±28.1	9.2±6.9
	Gr.1	No.30+control	23	32	157	212±24.6***	14.2±6.5
		No.40+control	35	50	215	300±39.0	7.1±1.6
		No.57+control	18	38	69	125±24.0***	13.7±5.5
	Gr.3	No.71+control	25	50	130	206±18.8***	6.2±0.3
		No.80+control	12	19	110	141±34.8***	9.6±4.3

* Stages 1 and 2, root nodule primordia;

Stage 3, mature root nodules; ND, not determined; NO, not observed; and DAI, days after inoculation with rhizobia. Dunnett test comparisons with control, n=3,

* *P*<0.05,

*** *P*<0.001.

At 15 DAI, the ARA was calculated as C_2_H_4_ (μmol h^−1^ g^−1^ nod. F.W.).
