# Supplementary material for: Evaluation of Immune Responses Induced by Simultaneous Inoculations of Soybean (Glycine max [L.] Merr.) with Soil Bacteria and Rhizobia
Source: Microbes Environ. 2019 Feb 5;34(1):64–75. doi: 10.1264/jsme2.ME18110 (PMC6440728; doi:10.1264/jsme2.ME18110)
Supplement: Supplementary file 1 [file 34_64_s1.pdf]

Evaluation of immune responses induced by simultaneous inoculations of soybean (*Glycine max* [L.] Merr.) with soil bacteria and rhizobia

Sayed Ziauddin Hashami[1], Hiroyuki Nakamura[2], Naoko Ohkama-Ohtsu[3], Katsuhiro Kojima[2], Salem Djedidi[2], Izumi Fukuhara[2], Mohammad Daud Haidari[5], Hitoshi Sekimoto[4], and Tadashi Yokoyama[3]*

Supplementary Materials

Table S1: Primers sets used for real-time PCR

Primer name	Primer Nucleotide Sequences	Reference
*SUBI-2*f	5'AGCTATTCGCAGTTCCCAAAT3'	Yasuda et al., 2016
*SUBI-2*r	5'CAGAGACGAACCTTGAGGAGA3'	Yasuda et al., 2016
*PR-1*f	5'AACTATGCTCCCCCTGGCAACTATATTG3'	Kwon and Ricke 2000
*PR-1*r	5'TCTGAAGTGGCTTCTACATCGAAACAA3'	Kwon and Ricke 2000
*PR-2*f	5'TGAGAGTGGATGGCCTTCTT3'	Kwon and Ricke 2000
*PR-2*r	5'TGTTTCACATTCCGAACCAA3'	Kwon and Ricke 2000
*PR-5*f	5'GTGAGCGACCTGAACACCAT3'	Yasuda et al., 2016
*PR-5*r	5'GCAGTACTTGGGGGAGTTGA3'	Yasuda et al., 2016
*PDF1.2*f	5'CATACAGGGGTCCATGCTTC3'	Yasuda et al., 2016
*PDF1.2*r	5GCATCTGCCTCTGAGCAAGT3'	Yasuda et al., 2016

*f: forward, r: reverse

Table S2. RT-PCR analyses of plant defense-related genes (*PR-1*, *PR-2*, *PR-5*, and *PDF1.2*) in the roots of the soybean mutant NOD1-3 under single inoculation of *Bradyrhizobium diazoefficiens* USDA 110^T and Group (Gr) 1 or 3 soil bacteria.

	Treatments	Gene	Sampling time (d)				
			2 DAS	5 DAS	7 DAS	10 DAS	20 DAS
	Un-inoculated (Ctr1)	*PR-1*	1	1	1	1	1
	USDA 110^T (Ctr2)	*PR-1*	1	8***	2.1	3.2	1.3
Gr.1 bacteria	No.23	*PR-1*	1	6	4.3	1.1	0.6
	No.30	*PR-1*	6	17*	2.5	0.3	0.3
	No.40	*PR-1*	3	6*	0.5	1	0.2
Gr.3 bacteria	No.57	*PR-1*	2	40*	2.7	1.2	1.1
	No.71	*PR-1*	3	107*	1.3	0.6	0.7
	No.80	*PR-1*	2	21***	1.3	1.7	0.8
	Un-inoculation (Ctr1)	*PR-2*	1	1	1	1	1
	USDA 110^T (Ctr2)	*PR-2*	1.7	5	1	1.1	0.5
Gr.1 bacteria	No.23	*PR-2*	0.2	8*	0.8	0.6	0.7
	No.30	*PR-2*	0.5	10	1.1	0.2	0.1
	No.40	*PR-2*	0.4	8*	0.3	0.1	0.2
Gr.3 bacteria	No.57	*PR-2*	0.6	9	2.7	0.5	1
	No.71	*PR-2*	0.3	20	0.5	0.1	0.5
	No.80	*PR-2*	0.8	4**	1.1	0.3	0.7
	Un-inoculated (Ctr1)	*PR-5*	1	1	1	1	1
	USDA 110^T (Ctr2)	*PR-5*	1.3	1.5	1	2.1	0.4
Gr.1 bacteria	No.23	*PR-5*	0.8	0.8	2.1	0.8	0.2
	No.30	*PR-5*	0.6	1.9	0.9	0.5	0.1
	No.40	*PR-5*	0.7	5**	0.3	0.4	0.2
Gr.3 bacteria	No.57	*PR-5*	0.3	3.5*	1.3	0.5	0.4
	No.71	*PR-5*	0.1	6.5*	0.9	0.4	0.3
	No.80	*PR-5*	0.6	2.2*	0.7	0.4	0.2
	Un-inoculated (Ctr1)	*PDF1.2*	1	1	1	1	1
	USDA 110^T (Ctr2)	*PDF1.2*	0.17	3	1.6	2.4	1.2
Gr.1 bacteria	No.23	*PDF1.2*	0.04	1.1	0.7	2.7	2.4
	No.30	*PDF1.2*	0.02	1.3	1.9	1.9	3.6
	No.40	*PDF1.2*	0.02	1	0.8	1.2	2.8
Gr.3 bacteria	No.57	*PDF1.2*	0.1	5.4*	4.4	3.9	6.8
	No.71	*PDF1.2*	0.35	9.1*	8.5	2.9	7.4
	No.80	*PDF1.2*	0.15	8*	6.9	6	5.8

Ctr1, treatment without bacterial inoculation; and Ctr2, USDA 110^T only inoculation. The expression level of each gene was normalized to the *SUBI-2* (ubiquitin) gene. Means ± standard deviations of three biological replicate are shown as 1 in the mean of the control (Ctr1) condition. Statistical analyses (Dunnett-test, n=3,**P* < 0.05, ***P* < 0.01, *** P <0.001) were performed for comparisons with Ctr1.

Table S3. RT-PCR analysis of plant defense-related genes *(PR-1*, *PR-2*, *PR-5*, and *PDF1.2*) in the roots of the soybean mutant NOD1-3 after being co-inoculated with *Bradyrhizobium diazoefficiens* USDA 110^T plus individual Group (Gr) 1 or 3 soil bacteria.

	Treatments	Gene	Sampling time (d)		
			7 DAS	10 DAS	20 DAS
	Un-inoculated (Ctr1)	*PR-1*	1	1	1
	USDA 110^T (Ctr2)	*PR-1*	2.1	3.2*	1.3
Gr.1 bacteria	No.23+Ctr2	*PR-1*	1.2	4.4	2.3
	No.30+Ctr2	*PR-1*	2.1	2	2.3
	No.40+Ctr2	*PR-1*	1.2	1.6	1.6
Gr.3 bacteria	No.57+Ctr2	*PR-1*	4.9*	1.2	2.8
	No.71+Ctr2	*PR-1*	2.2	1.5	4.7*
	No.80+Ctr2	*PR-1*	1.1	3	2.8
	Un-inoculation (Ctr1)	*PR-2*	1	1	1
	USDA 110^T (Ctr2)	*PR-2*	1	1.1	0.5
Gr.1 bacteria	No.23+Ctr2	*PR-2*	0.5	0.4	0.2
	No.30+Ctr2	*PR-2*	0.8	0.3	0.2
	No.40+Ctr2	*PR-2*	0.6	0.7	0.1
Gr.3 bacteria	No.57+Ctr2	*PR-2*	2.7	0.2	0.8
	No.71+Ctr2	*PR-2*	1.9	0.4	0.2
	No.80+Ctr2	*PR-2*	0.4	0.1	0.3
	Un-inoculated (Ctr1)	*PR-5*	1	1	1
	USDA 110^T (Ctr2)	*PR-5*	1	2.1	0.4
Gr.1 bacteria	No.23+Ctr2	*PR-5*	1.2	1.4	0.1
	No.30+Ctr2	*PR-5*	0.7	1	0.1
	No.40+Ctr2	*PR-5*	0.7	0.8	0.1
Gr.3 bacteria	No.57+Ctr2	*PR-5*	0.9	0.5	0.4
	No.71+Ctr2	*PR-5*	0.7	0.8	0.1
	No.80+Ctr2	*PR-5*	0.5	1.3	0.2
	Un-inoculated (Ctr1)	*PDF1.2*	1	1	1
	USDA 110^T (Ctr2)	*PDF1.2*	1.6	2.4	1.2
Gr.1 bacteria	No.23+Ctr2	*PDF1.2*	0.2	1.8	1.5
	No.30+Ctr2	*PDF1.2*	0.2	2.8	1.3
	No.40+Ctr2	*PDF1.2*	0.3	1.2	1.1
Gr.3 bacteria	No.57+Ctr2	*PDF1.2*	1.6	3.7*	3*
	No.71+Ctr2	*PDF1.2*	3.6*	4.4**	5.1
	No.80+Ctr2	*PDF1.2*	3.6*	3.5*	4.5*

Ctr1, treatment without bacterial inoculation; and Ctr2, USDA 110^T only inoculation. The expression level of each gene was normalized to the *SUBI-2* (ubiquitin) gene. Means ± standard deviations of three biological replicate are shown as 1 in the mean of the control (Ctr1) condition. Statistical analyses (Dunnett-test, n=3,*P < 0.05, **P < 0.01) were performed for comparisons with Ctr1.
